# The Proteomic Landscape of Parkin-Deficient and Parkin-Overexpressing Rat Nucleus Accumbens: An Insight into the Role of Parkin in Methamphetamine Use Disorder

**DOI:** 10.3390/biom15070958

**Published:** 2025-07-03

**Authors:** Akhil Sharma, Tarek Atasi, Florine Collin, Weiwei Wang, TuKiet T. Lam, Rolando Garcia-Milian, Tasnim Arroum, Lucynda Pham, Maik Hüttemann, Anna Moszczynska

**Affiliations:** 1 Department of Pharmaceutical Sciences, Wayne State University, Detroit, MI 48202, USA; akhilsharma@wayne.edu (A.S.);; 2 Department of Molecular Biophysics and Biochemistry, Yale University, New Haven, CT 06520, USA; florine.collin@yale.edu (F.C.); weiwei.wang@yale.edu (W.W.); tukiet.lam@yale.edu (T.T.L.); 3 Keck Mass Spectrometry & Proteomics Resource, Yale School of Medicine, New Haven, CT 06520, USA; 4 Yale/NIDA Neuroproteomics Center, Yale School of Medicine, New Haven, CT 06520, USA; 5 Harvey Cushing/John Hay Whitney Medical Library, Yale University, New Haven, CT 06520, USA; rolando.milian@yale.edu; 6 Center for Molecular Medicine and Genetics, Wayne State University, Detroit, MI 48201, USA; ho0066@wayne.edu (T.A.); lucyndapham@wayne.edu (L.P.); mhuttema@med.wayne.edu (M.H.)

**Keywords:** parkin, proteome, nucleus accumbens, mitochondria, methamphetamine

## Abstract

In recent years, methamphetamine (METH) misuse in the US has been rapidly increasing, and there is no FDA-approved pharmacotherapy for METH use disorder (MUD). We previously determined that ubiquitin-protein ligase parkin is involved in the regulation of METH addictive behaviors in rat models of MUD. Parkin is not yet a “druggable” drug target; therefore, this study aimed to determine which biological processes, pathways, and proteins downstream of parkin are likely drug targets against MUD. Employing young adult Long Evans male rats with parkin deficit or excess in the nucleus accumbens (NAc), label-free proteomics, and molecular biology, we determined that the pathways downstream of parkin that are candidates for regulating METH addictive behaviors in young adult male rats are mitochondrial respiration, oxidative stress, AMPA receptor trafficking, GABAergic neurotransmission, and actin cytoskeleton dynamics.

## 1. Introduction

In the United States, there are close to 2,000,000 people who use methamphetamine (METH), and deaths from METH overdose are rapidly rising [1,2]. Approximately 30% of people who use METH develop METH use disorder (MUD). MUD is a chronic, relapsing disorder that has been characterized by compulsive seeking and escalated intake of METH. Despite numerous clinical trials, there is no FDA-approved pharmacotherapy for MUD. Furthermore, genetic susceptibility to MUD is poorly understood. A recent systematic review and meta-analysis of human gene association studies identified several promising gene targets in MUD [3]. Our group demonstrated in a rat model of MUD that the *parkin* gene may also be such a target. Specifically, our previous study in a rat model of MUD showed that total body parkin knockout was associated with increased self-administration of METH and METH-induced conditioned place preference. In contrast, overexpression of parkin in the nucleus accumbens (NAc) before METH exposure had the opposite effect on these addictive behaviors [4]. These results suggest that parkin-deficient rats (*Park2^−/−^*, PKO rats) are more vulnerable to developing MUD compared to their wild-type (WT) counterparts, while rats overexpressing parkin in the NAc (PO rats) are less predisposed. Parkin is a component of the ubiquitin-proteasome system and plays a critical role in maintaining mitochondrial function, including protection from various cellular stresses [5,6]. In addition to regulating energy metabolism, parkin has also been linked to glutamatergic and dopaminergic neurotransmission, cytoskeleton dynamics, oxidative stress, and inflammatory responses [5,7,8,9,10,11,12,13,14,15,16,17,18]; all these processes have been implicated in MUD [19,20,21,22,23,24,25,26,27,28,29]. To date, most studies investigated parkin’s role in Parkinson’s disease and other neurodegenerative diseases. Moreover, no studies analyzed the proteomic landscape from parkin-deficient or parkin-overexpressing NAc, a reward-mediating brain area [30]. We were the first to demonstrate that parkin plays a role in MUD, and this investigation is the first to generate and analyze proteomes from PKO and PO NAc to gain insight into the potential mechanisms involved in the anti-addictive properties of parkin in MUD.

## 2. Materials and Methods

### 2.1. Animals

**Figure 1:**
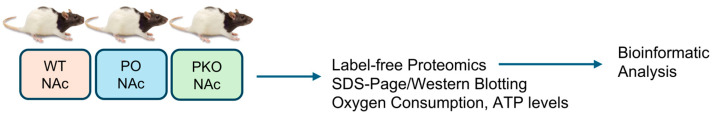
Experimental Design. Nucleus accumbens tissue from wild-type (WT), parkin knockout (PKO), and parkin-overexpressing (PO) rats was assessed for proteomic landscape and the levels of several Krebs cycle and electron transport chain enzymes by SDS-PAGE/western blotting. Bioinformatic analyses were then used to identify biological processes impacted by parkin deficit or excess in the NAc. The tissues were also assessed for oxygen consumption rate and ATP levels.

All experiments and procedures were approved by the Wayne State University Institutional Animal Care and Use Committee (protocol # 22-09-4982) and conducted in compliance with all standards for animal care and investigation established in the Guide for the Care and Use of Laboratory Animals (National Research Council 2011) and the Federal Animal Welfare Act. The study employed N = 36 adult male Long Evans rats: WT, PKO (*Park2^−/−^*), and PO (~67 days old at the beginning of the experiment) acquired from Inotiv (previously Envigo, Indianapolis, IN, USA) and Charles River Laboratories (Willington, MA, USA). The PO rats were generated in our laboratory by bilaterally overexpressing parkin in the NAc, as described below. All rats were allowed to acclimate for one week after delivery. The rats were housed in a temperature- and humidity-controlled room under a 12:12 h light/dark cycle (temperature: ~21 °C; humidity 40–70%). The rats were sacrificed by decapitation one month after their arrival. NAc tissue was dissected and subjected to several analyses (Figure 1). Three rats per group were used for the proteomic analysis, *n* = 4 per group for measurement of mitochondrial function, and *n* = 7 per group were employed in SDS-PAGE and western blotting. Parkin knockout and parkin overexpression were validated by SDS-PAGE and western blotting).

### 2.2. Parkin Overexpression Employing the Adeno-Associated Viral Transfer Vector

The adeno-associated viral vector 2/6 (AAV2/6-parkin) was produced and titrated, as described previously [31], in the laboratory of Dr. Bernard Schneider at the Bertarelli Foundation Gene Therapy Core Facility, Swiss Federal Institute of Technology (EPFL), Geneva, Switzerland. The stereotaxic surgery was performed as previously published [4]. Briefly, rats were anesthetized with 4% isoflurane (Primal Critical Care, Bethlehem, PA, USA) and maintained under anesthesia at 2% isoflurane. AAV2/6-parkin was bilaterally injected at a 16° angle into the NAc with the following coordinates: +1.8 A/P, ±3.2 M/L, −7.6 D/V from Bregma, according to the rat brain atlas of Paxinos and Watson, at the amount of 6.5 × 10^7^ TUs/hemisphere in a volume of 1.6 μL. Viral suspensions were injected with a 10-μL Hamilton syringe at a rate of 0.15 μL/min using a syringe pump (Harvard Apparatus, Holliston, MA, USA). The syringe was left in place for 5 min at −7.6 mm, then withdrawn to −6.6 mm and −5.5 mm, staying in each location for 5 min, then raised slowly out of the brain over 5 min. The rats were sacrificed 3 weeks after the surgery to allow for maximal parkin overexpression.

### 2.3. Tissue Collection and Sample Preparation

NAc tissue was harvested from a 2 mm thick coronal brain section encompassing 0.7–2.7 mm from Bregma using a 1.5 mm biopsy plunger (Kai Corporation, Osaka, Japan), flash frozen on dry ice, weighted, and stored at −80 °C until assayed. All analyses used tissue from individual animals as distinct data points. There was no pooling of samples between animals.

#### 2.3.1. LC-MS/MS

For proteomic analysis, frozen NAc pieces were submitted to the Keck Mass Spectrometry (MS) & Proteomics Resource at the Yale School of Medicine for sample preparation and analysis. The NAc from each rat (~6 mg, *n* = 3 rats/group) was sonicated in 400 µL of ice-cold RIPA buffer (50 mM Tris, pH 8.0, 1 mM EDTA, 1% Triton X-100, 0.1% sodium deoxycholate, 0.1% SDS, and 110 mM NaCl) containing Halt Protease and Phosphatase Inhibitor Cocktails (Fisher Scientific, Waltham, MA, USA), 50 µM proteasome inhibitor MG132, and 50 µM deubiquitinases inhibitor PR-619 (MilliporeSigma, Burlington, MA, USA). Samples were subjected to two 15-s rounds of sonication (10% amplitude, 1-s on-and-off pulses) on a cooling rack using a probe sonicator (Fisher Scientific, Waltham, MA, USA). The sonicate was centrifuged at 14,600× *g* for 10 min at 4 °C. An aliquot of 100 µL of the supernatant was taken, and proteins were precipitated utilizing a methanol/chloroform/water precipitation procedure: 400 μL of cold methanol was added to the aliquot and vortexed; 100 μL of chloroform was added and vortexed; and finally, 300 μL of water was added and vortexed. The resultant mixture was centrifuged at 14,600× *g* for 2 min at 4 °C. The top layer was removed and discarded. Four hundred μL of cold methanol was added to the remaining mixture, vortexed, and centrifuged at 14,600× *g* for 2 min at 4 °C. The supernatant was removed and discarded. The cold methanol wash was repeated one more time. The precipitated protein pellet was allowed to air dry for one minute and resuspended in 80 µL of 8 M urea containing 400 mM ABC (ammonium bicarbonate), reduced with DTT (8 µL of a 45 mM stock solution, incubated for 30 min at 37 °C and cooled to room temperature), alkylated by the addition of 8 μL iodoacetamide (100 mM), and then diluted with 184 µL H_2_O before enzymatic digestion by incubation with LysC (40 µL of a 0.1 µg/µL stock solution, incubated at 37 °C overnight). Eight µL of a 0.5 µg/µL stock trypsin solution was then added and allowed to digest for 6 h at 37 °C. Digestion (total volume of 320 µL) was quenched with 16.4 µL of 20% TFA and stored at −20 °C until a desalting step using C18 MacroSpin columns (The Nest Group, Ipswich, MA, USA). The effluents from the desalting step were dried and re-dissolved in 50 µL of loading buffer (LB) solution (98% H_2_O, 2% acrylonitrile [ACN], and 0.2% trifluoroacetic acid [TFA]). An aliquot was taken, concentration measured via Nanodrop, and diluted to 0.06 µg/µL with LB. Next, 1:4 dilution of 10× Pierce Retention Time Calibration Mixture (cat #88321; Thermo Fisher Scientific, Waltham, MA, USA) was added to each sample *prior* to injecting on the UPLC Q-Exactive HFX mass spectrometer (Thermo Fisher Scientific, Waltham, MA, USA) for LFQ data collection. The RTCalMix was spiked into the sample as an instrument’s internal quality control check for retention time variability and utilized for normalization during LFQ data analysis.

#### 2.3.2. SDS-PAGE and Western Blotting

SDS-PAGE was performed on mitochondrial and cytosolic fractions. To isolate crude NAc mitochondria, 5–7 mg of NAc tissue (*n* = 4 rats/group) was homogenized with pre-chilled KIMBLE^®^ KONTES^®^ Potter-Elvehjem Tissue Grinder with PTFE pestle (cat# 886000-0018, DWK Life Sciences, Vineland, NJ, USA) and then processed according to the manufacturer instructions (Mitochondria Isolation Kit for Tissues; cat# 89801, Thermo Fisher Scientific, Waltham, MA, USA). Protein concentrations in cytoplasmic and mitochondrial fractions were estimated using the BCA assay. The purity of isolated mitochondria was ascertained by SDS-PAGE and western blotting using mitochondria-specific antibodies (Supplementary Figure S1).

#### 2.3.3. Complex IV Activity and ATP Levels

To investigate the impact of parkin overexpression or knockout on the kinetics of complex IV activity, the rate-limiting step in the mitochondrial electron transport chain, each of two NAc tissue pieces (1–3 mg) was immersed in ice-cold 500 µL of a solubilization buffer consisting of 10 mM K-HEPES (pH 7.4), 40 mM KCl, 1% Tween 20, 1 mM phenylmethylsulfonyl fluoride, 1 mM heat-activated sodium vanadate, 10 mM KF, and 2 mM EGTA. The tissue was then homogenized using a ultrasonicator (Fisher Scientific, Waltham, MA, USA), by applying two sets of 8-s pulses. Protein concentrations in both cytoplasmic and mitochondrial fractions were estimated using the BCA assay. One piece was used to measure complex IV activity, and the other was used to measure ATP levels. The NAc tissue pieces (*n* = 4 rats/group) were from different PKO, PO, and WT rats than those assessed by LC-MS/MS and/or SDS-PAGE and western blotting.

### 2.4. Label-Free Quantification (LFQ) Data Collection

LFQ data-dependent acquisition (DDA) was performed on a Thermo Scientific Q-Exactive HFX mass spectrometer connected to a Waters M-Class ACQUITY UPLC system (Waters Corporation, Milford, MA, USA) equipped with a Waters Symmetry^®^ C18 180 μm × 20 mm trap column and a 1.7-μm, 75 μm × 250 mm nanoACQUITY UPLC column (35 °C). Five µL of each digest were reconstituted in Loading Buffer A to a concentration of 0.05 µg/µL and were injected in block-randomized order. UPLC peptide trapping was carried out for 3 min at 10 µL/min in 99% Buffer A (0.1% FA in water) and 1% Buffer B [(0.1% FA in acetonitrile (ACN)] prior to eluting with linear gradients that started at 3% B and reached 6% B at 2 min, 25% B at 200 min, 85% B at 205 min, and stayed at 85% for 5 min prior to dropping back to 3% B at 212 min. Three blanks (1st with 50% ACN, 2nd and 3rd with Buffer A) followed each injection to ensure against sample carryover. Settings for the Q-Exactive HFX mass spectrometer (Thermo Fisher Scientific, Waltham, MA, USA) included: 60,000 MS scan resolution with AGC target of 3 × 10^6^ (Max IT of 100 ms) and a scan range of 350–1500 *m/z* in profile mode; 30,000 MS2 scan resolution with AGC target of 1 × 10^5^ (Max IT of 100 ms) and a scan range of 200–2000; and Top20 peptide HCD fragmentation consist of isolation window of 1.6 *m/z*, normalized collision energy of 28, peptide match preferred with all multiple charge state, and dynamic exclusion of 20 s. All MS and MS/MS peaks were detected in the Orbitrap.

Protein abundances and their accession number were then utilized in Gene Enrichment Set Analysis (GSEA) [32], ShinyGO v.8.2 [33], and Ingenuity Pathway Analysis (IPA) (Qiagen, Redwood City, CA, USA) for determination of differentially expressed proteins (DEPs) between experimental groups and biological processes and pathways to which the DEPs belong.

### 2.5. SDS-PAGE and Western Blotting

Mitochondrial protein levels were assessed in protein extracts leftover from the proteomic analysis (*n* = 3 rats/group) and crude mitochondria from nine additional rats (*n* = 4 rats/group). Of note, the antibodies against mitochondrial enzymes did not bind to non-specific proteins in the cytoplasm (Supplementary Figure S1). The protein extracts and crude mitochondria were denatured using undiluted lithium dodecyl sulfate (1 × LDS) sample buffer (Invitrogen Life Technologies, Carlsbad, CA, USA) containing 5% β-mercaptoethanol and incubated at 70 °C for 10 min. Samples of 10–20 μg of protein were loaded per lane and resolved on 4–12% precast gradient Bis-Tris gels (Invitrogen Life Technologies, Carlsbad, CA, USA). Proteins were transferred to PVDF membrane (EMD Millipore, Burlington, MA, USA) at 400 mA, blocked with 5% non-fat dried milk dissolved in TBST (10 mM Tris, 150 mM NaCl, and 0.05% Tween-20), and incubated overnight at 4 °C with the following primary antibodies (diluted in 5% milk-containing TBST) against the following proteins: citrate synthase (CS) (NBP2-13878, 1:1000 Novus Biologicals, Centennial, CO, USA), dihydrolipoamide S-succinyltransferase (DLST) (PA5-51794, 1:1000,Invitrogen Life Technologies, Carlsbad, USA), mitochondrial malate dehydrogenase (MDH2) (9610, 1:1000, Cell Signaling, Danvers, MA, USA), dihydrolipoamide dehydrogenase (DLD) (NBP1-31302, 1:1000, Novus Biologicals, Centennial, CO, USA), 2-oxoglutarate dehydrogenase (OGDH) (15212-1-AP, 1:500, Proteintech Group Inc., Rosemont, IL, USA), mitochondrial complex I (mouse anti-NDUFS3 subunit, ab110246, 1: 2000, Abcam Inc., Waltham, MA, USA), complex II (mouse anti-SDHA subunit, ab14715, 1:2500, Abcam Inc., Waltham, MA, USA), complex III (mouse anti-UQCRC2 subunit, ab14745, 1:1000, Abcam Inc., Waltham, MA, USA), complex IV (rabbit anti-subunit IV, 4844, 1:1000, Cell Signaling Technology, Danvers, MA, USA), ß-actin (ab8227, 1:1000, Abcam Inc., Waltham, MA, USA), and glyceraldehyde 3-phosphate dehydrogenase (GAPDH) (2174, Cell Signaling Technology, 1:2000, Danvers, MA, USA). The incubation with primary antibodies was followed by washing with TBST (three times for 5 min each) followed by incubation with an appropriate horseradish peroxidase-conjugated secondary antibodies (1:10,000, 60 min at room temperature). Blots were developed using enhanced chemiluminescence detection and LAS4000 bioimager (GE Healthcare, Chicago, IL, USA). To reduce bias, each blot contained all experimental groups. The western blot data were expressed as relative optical density units on each gel normalized to WT controls. This approach normalized differences in the development of the blots. Original figures can be found in Supplementary Materials. 

### 2.6. Complex IV-Specific Activity and ATP Levels

The analysis of oxygen consumption was conducted in the presence of 20 mM ascorbate and 30 µM cytochrome c (Sigma-Aldrich, Saint Louis, MO, USA, cat # C3131). Continuous monitoring of oxygen consumption rates allowed for data collection in nmol/min/mg of total protein, which was subsequently analyzed using OxyTrace+ software (version 1.0 Build 48, 2016, Hansatech Instruments Ltd., Amesbury, MA, USA) [34,35]. It should be noted that this protocol avoids the use of compounds such as N,N,N′,N′-tetramethyl-para-phenylene-diamine (TMPD) and the detergent dodecyl maltoside (DDM) because they render the complex IV kinetics unphysiological as extensively discussed [36,37]. ATP levels were assessed using the boiling method on flash-frozen tissue samples with the ATP bioluminescence assay kit HS II (Roche Applied Science, Penzberg, Germany), and data were standardized based on protein concentration and normalized to the WT group as described previously [34,35].

### 2.7. Data Analyses

#### 2.7.1. LFQ Data Analysis

The LC-MS/MS data was processed with Progenesis QI software (Nonlinear Dynamics, version 4.2), with protein identification carried out using the in-house Mascot search engine (2.8). The Progenesis QI software performs chromatographic/spectral alignment (one run is chosen as a reference for alignment of all other data files to it), mass spectral peak picking and filtering (ion signal must satisfy 3 times the standard deviation of the noise), and quantitation of proteins and peptides. A normalization factor for each run was calculated to account for differences in sample load between injections as well as differences in ionization. The normalization factor was determined by calculating a quantitative abundance ratio between the reference run and the run being normalized, with the assumption being that most proteins/peptides are not changing in the experiment, such that the quantitative value should equal 1. The experimental design was set up to group multiple injections (biological replicates) from each run into each comparison set. The algorithm then calculated the tabulated raw and normalized abundances, as well as the ANOVA *p*-values for each feature in the dataset. The MS and MS/MS spectra (after filtering for the top 10 MS/MS spectra per selected MS feature) were exported as .mgf (Mascot generic files) for database searching. Mascot search algorithm was used to search against the Swiss Protein database, with taxonomy restricted to *Rattus norvegicus*. The carbamidomethyl (Cys), oxidation (Met), phosphorylation (Ser, Thr, Tyr), deamidation (NQ), acetylation (K, Protein N-Term), and ubiquitinylation (K) were entered as variable modifications. Two missed tryptic cleavages were allowed, precursor mass tolerance was set to 10 ppm, and fragment mass tolerance was set to 0.02 Da. The significance threshold was set based on a False Discovery Rate (FDR) of 2% with a 95% MOWSE score of confidence level. The Mascot search results were exported as .xml files and then imported into the processed dataset in Progenesis QI software, where peptide IDs were synched with the corresponding quantified features and their corresponding abundances. Protein abundances (requiring at least one unique peptide with scores above the significant threshold) were then calculated from the sum of all unique normalized non-conflicting peptide ions for a specific protein on each run. Resulting LFQ data were exported, and comparative analyses were calculated in Excel.

Qlucore Omics Explorer (Qlucore, Lund, Sweden) was used for data analysis and visualization, including unsupervised Principal Component Analysis (PCA), hierarchical clustering heatmaps, and volcano plots, with *p* < 0.01 or *p* < 0.05 used for DEP selection. Overrepresented pathways were identified using GSEA software (v. 4.3.3) [32], which utilized predefined gene sets from the Molecular Signatures Database (MSigDB v. 2024.1, Broad Institute and UC San Diego). A gene set is a group of genes that share pathways, functions, chromosomal localization, or other features. For the present study, we used C1 and C5 collection sets for GSEA analysis. We leveraged the human MSigDB collection based on the evolutionary conservation of key signaling pathways between humans and rats. The following parameters were calculated: the enrichment score (ES), absolute enrichment score (abs (ES)), normalized enrichment score (NES), false discovery rate (FDR), and nominal *p*-value (see [32] for more details). The ES reflects the degree to which a gene set is overrepresented at the top or bottom of a ranked list of genes. GSEA calculates the ES by walking down the ranked list of genes, increasing a running-sum statistic when a gene is in the gene set and decreasing it when it is not. The magnitude of the increment depends on the gene’s correlation with the phenotype. The ES is the maximum deviation from zero encountered when walking the list. A positive ES indicates gene set enrichment at the top of the ranked list; a negative ES indicates the enrichment at the bottom. The normalized enrichment score (NES) accounts for differences in gene set size and correlations between gene sets and the expression dataset; therefore, the NES can be used to compare analysis results across gene sets. The false discovery rate (FDR *q*-value) in GSEA represents the estimated probability that a gene set with a given normalized enrichment score (NES) represents a false positive finding. The nominal *p*-value estimates the statistical significance of the enrichment score for a single gene set. Gene sets with *p* < 0.05 were considered significantly enriched. The ShinyGo (v.8.2) software examined biological pathways with an FDR cutoff of 0.05 and pathway size of 2–5000. The IPA software (QIAGEN, v. 134816949) was used to identify upstream transcriptional regulators, setting the *p*-value at 0.05.

#### 2.7.2. Molecular Biology Data Analysis

Densitometry data sets were analyzed by one-way ANOVA with Holm-Sidak’s post hoc test, while correlations were assessed by Pearson’s correlation analysis, employing GraphPad Prism v.10.4.1. Statistical significance was set at *p* < 0.05. The data is expressed as mean ± SEM.

## 3. Results

### 3.1. Differentially Expressed Proteins in Rat Nucleus Accumbens Lacking Parkin

**Figure 2:**
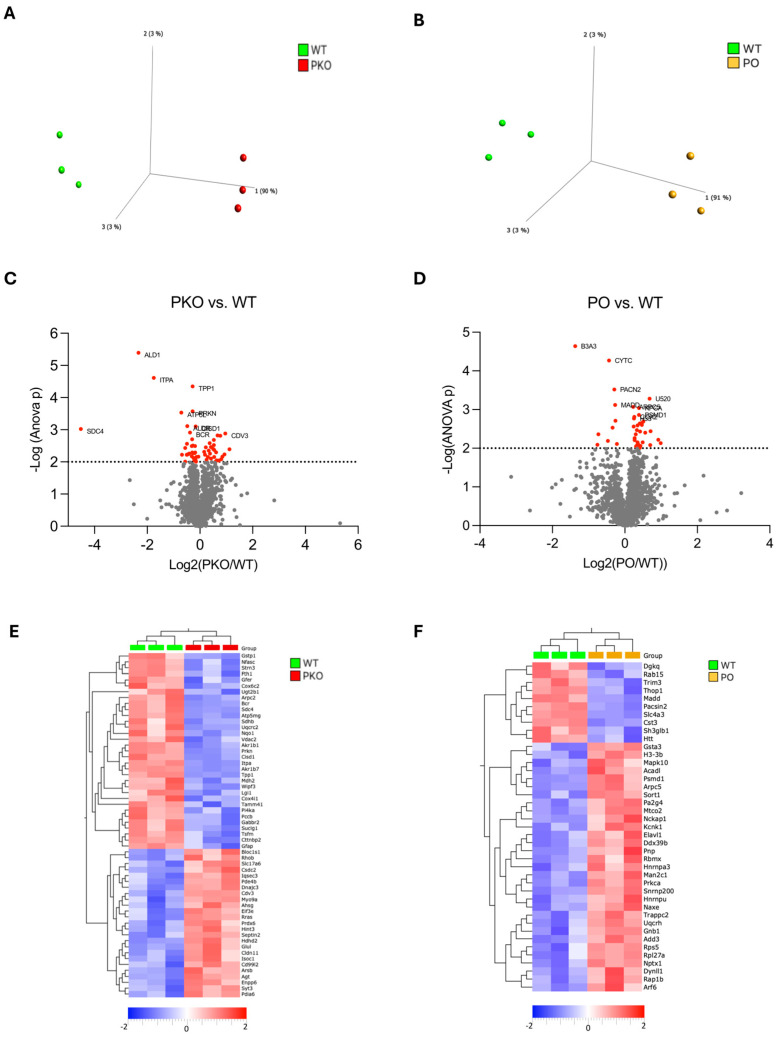
Differentially Expressed Proteins in Parkin-Deficient and Parkin-Overexpressing Rat Nucleus Accumbens. (**A**,**B**) Principal component analysis (PCA) of proteomic data derived from parkin-deficient (PKO), parkin-overexpressing (PO), and wild-type (WT) rat nucleus accumbens (n = 3/group). Each mark represents an individual rat. Note that the PKO and PO groups are clearly separate from the WT group. (**C**,**D**) Volcano plots showing relationships between the magnitude of change in protein expression (log2 of fold change; *x*-axis) and statistical significance of this change (−log10(*p*); *y*-axis] in a comparison of PKO to WT and PO to WT. Colored points represent differentially expressed proteins (DEPs) at a cutoff *p* = 0.01. (**E**,**F**) Heatmaps depicting DEPs upregulated (red) and downregulated (blue) in rat nucleus accumbens by parkin knockout (PKO) or overexpression (PO) relative to wild-type (WT) nucleus accumbens (*p* < 0.01, Qlucore Explorer).

A total of 2247 proteins were detected by label-free proteomics when PKO was compared to WT rats (see Supplementary Table S1 for the complete list). The principal component analysis (PCA) plot shows a clear separation between PKO and WT groups (Figure 2A). We identified 58 differentially expressed proteins (DEPs) in PKO NAc (*p* < 0.01, ANOVA): 22 upregulated and 36 downregulated. These DEPs are presented in the Volcano plot (Figure 2C, red dots), heatmap (Figure 2E), and Supplementary Material (Table S2). Nine of the top ten DEPs were downregulated: aldo-keto reductase family 1 member B7 protein (ALD1), inosine triphosphatase pyrophosphohydrolase (ITPA), tripeptidyl peptidase 1 (TPP1), ATP synthase membrane subunit G (ATP5L), aldo-keto reductase family 1 member B1 (ALDR), CDGSH iron-sulfur domain-containing protein 1 (CISD1), breakpoint cluster region protein (BCR), syndecan-4 (SDC4), parkin (PRKN), and one protein, protein CDV3 homolog (CDV3), was upregulated. The DEPs that most dramatically changed their abundance upon parkin knockout were ALD1 (−80%), ITPA (−80%), SDC4 (−95%), and BCR (+95%).

### 3.2. Differentially Expressed Proteins in Rat Nucleus Accumbens Overexpressing Parkin

A total of 2244 proteins were detected while PKO was compared to WT rats (see Supplementary Table S3 for the complete list). The PCA plot shows a clear separation between the PO and WT groups (Figure 2B). We identified 41 differentially expressed proteins (DEPs) in PO NAc (*p* < 0.01, ANOVA): 31 upregulated and 10 downregulated. These DEPs are presented in the Volcano plot (Figure 2D, red dots), heatmap (Figure 2), and Supplementary Material (Table S4). Six of the top ten DEPs were upregulated, and four were downregulated. The top six upregulated proteins were small nuclear ribonucleoprotein U5 subunit 200 (U520), actin-related protein2/3 (ARP) complex subunit 5 (ARPC5), protein kinase C α type (PRKCA), 26S proteasome non-ATPase regulatory subunit 1 (PSMD1), cytochrome c oxidase subunit 2 (COX2), and histone H3.3 (H33). The downregulated DEPs included solute carrier family 4 member 3 (B3A3), cystatin C (CYTC), protein kinase C and casein kinase substrate in neurons 2 (PACN2), and mitogen-activated protein kinase activating death domain (MADD). The DEPs most dramatically changed their abundance upon parkin overexpression were B3A3 (−61%) and U520 (+60%). Parkin was not one of the proteins significantly increased in abundance in the NAc of PO rats due to high variability in its levels.

### 3.3. Molecular Pathways Changed by Parkin Knockout or Parkin Overexpression in Rat Nucleus Accumbens

We employed Gene Set Enrichment Analysis (GSEA) to create protein enrichment maps. GSEA determines whether there is any chance that the group of genes being evaluated is in any way related to the phenotype. The analysis detects subtle enrichment patterns as opposed to IPA and other similar applications, which map significantly altered genes or proteins to curated pathways. Top enriched pathways and biological processes in the NAc of PKO rats compared to WT NAc (*p* < 0.01, *q* < 0.05) are presented in Table 1, with the complete list in the Supplementary Table S5. The most enriched pathways were those related to energy metabolism and pathways associated with neurodegenerative diseases.

Top enriched pathways and biological processes in the NAc of PO rats as compared to WT NAc (*q* < 0.05, *p* < 0.01) are presented in Table 2, with the complete list in the Supplementary Table S6. The most enriched pathways were those related to RNA processing and energy metabolism. It is worth noting that parkin might be involved in mRNA processing [38]; however, the enrichment in RNA pathways is most of an effect of the AAV. The list also included glutamatergic signaling via kainate receptors and Gβγ signaling through PLCβ (Table 2). NAc neurons produce mainly GABAergic, but functional presynaptic and postsynaptic kainite receptors are present in the NAc [39].

### 3.4. Molecular Pathways Changed by Parkin Knockout and Parkin Overexpression in Rat Nucleus Accumbens

We previously demonstrated that parkin knockout and overexpression of parkin in the NAc *prior* to METH exposure resulted in opposite changes in addictive behaviors, i.e., increased and decreased METH self-administration, respectively, as compared to WT controls [4]. Since PKO and PO rats displayed opposite changes in METH addictive behavior, we next examined which neuronal pathways and proteins were altered in opposite directions in the NAc of PKO and PO rats. GSEA revealed that pathways significantly changed in opposite directions in the NAc of PKO and PO rats compared to the WT controls (downregulated and upregulated, respectively) were energy metabolism pathways, regulation of the mitotic cell cycle, and GTPases-activated WASP and WAVES (Table 3). In mature neurons, proteins involved in the regulation of the mitotic cell cycle play a part in DNA repair and apoptosis [40]. The proteins belonging to the WASPS and WAVES family are actin cytoskeleton reorganizers [41].

**Figure 3:**
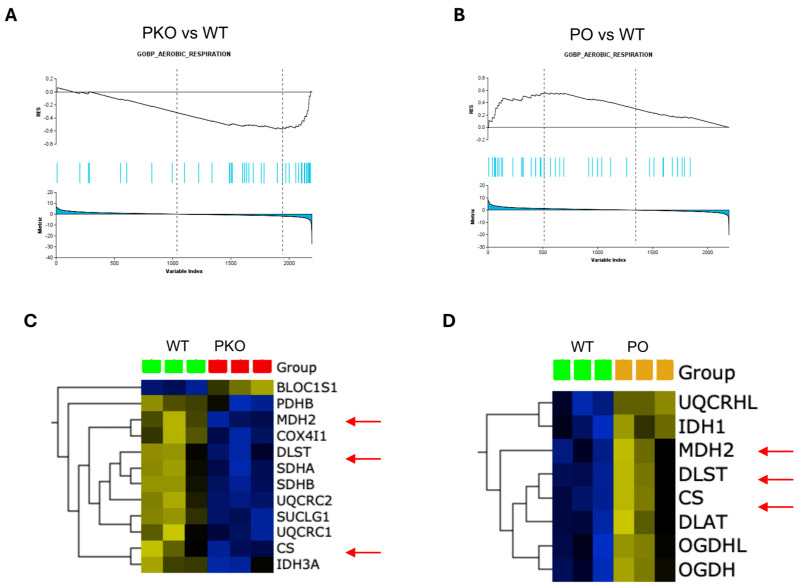
Parkin Deficit or Overexpression in Rat Nucleus Accumbens Changes Aerobic Respiration in Opposite Directions. (**A**,**B**) Gene set enrichment analysis (GSEA)-enrichment plots showing protein sets from GOBP_AEROBIC RESPIRATION signature significantly overrepresented (*p* < 0.05) in parkin knockout (PKO) or parkin overexpression (PO) rat nucleus accumbens, as compared to wild-type nucleus accumbens. (**C**,**D**) Hierarchical clustering heatmap of the leading-edge proteins from the GOBP_AEROBIC_RESPIRATION (*p* < 0.05) in PKO NAc and PO NAc. Three Krebs cycle enzymes, citrate synthase (CS), mitochondrial malonate dehydrogenase (MDH2), and dihydrolipoamide S-succinyltransferase (DLST), were decreased in PKO NAc and increased in PO NAc relative to wild-type NAc (red arrows).

As aforementioned, GSEA calculates an enrichment score (ES) that reflects the degree to which a gene or protein set is overrepresented at the extremes (top or bottom) of an entire ranked list of genes or proteins that differ between two phenotypes. The GSEA also identifies a subset of genes/proteins (leading-edge subsets) that impact the ES most. Figure 3 shows that proteins from the aerobic respiration signature are overrepresented at the top or bottom in PKO NAc and PO NAc, respectively, relative to WT NAc (Figure 3A,B). The hierarchical clustering heatmaps show the leading-edge proteins from the aerobic respiration signature (Figure 3C,D). Proteins significantly overrepresented (*p* < 0.05) in the aerobic respiration signature in PKO NAc included subunit beta of mitochondrial pyruvate dehydrogenase E1 (PDHB), a protein that links glycolytic pathway to the Krebs cycle, six Krebs cycle proteins (subunit α of succinyl-CoA synthetase (SUCLG1), subunit α of mitochondrial isocitrate dehydrogenase [NAD], (IDH3A), citrate synthase (CISI), mitochondrial malate dehydrogenase (MDH2), and dihydrolipoyllysine-residue succinyltransferase component of 2-oxoglutarate dehydrogenase complex (DLST), six electron transport chain (ETC) proteins: subiquinol-cytochrome C reductase core protein 2 (UQCRC2), cytochrome c oxidase subunit 4 isoform 1 (COX4I1), succinate dehydrogenase complex iron sulfur subunit B (SDHB), succinate dehydrogenase complex iron-sulfur subunit A (SDHA), ubiquinol-cytochrome C reductase complex III subunit VII (UQCRQ), ubiquinol-cytochrome C reductase core protein 1 (UQCRC1), and biogenesis of lysosome-related organelles complex 1 subunit 1, a component of the BLOC-1 complex that may negatively regulate aerobic respiration (UNIPROT). Proteins significantly overrepresented (*p* < 0.05) in the aerobic respiration signature in PO NAc included five Krebs cycle enzymes, CS, MDH2, DLST, 2-oxoglutarate dehydrogenase complex component E1 (OGDH), and 2-oxoglutarate dehydrogenase-like component (OGDHL) of the complex as well as cytochrome b-c1 complex subunit 6-like (UQCRHL) belonging to the ETC, and cytoplasmic isocitrate dehydrogenase [NADH] (IDH1). Among aerobic respiration DEPs, three Krebs cycle proteins were significantly altered in abundance in opposite directions in PKO and PO rats: CS, MDH2, and DLST (Figure 3C,D, arrows).

**Figure 4:**
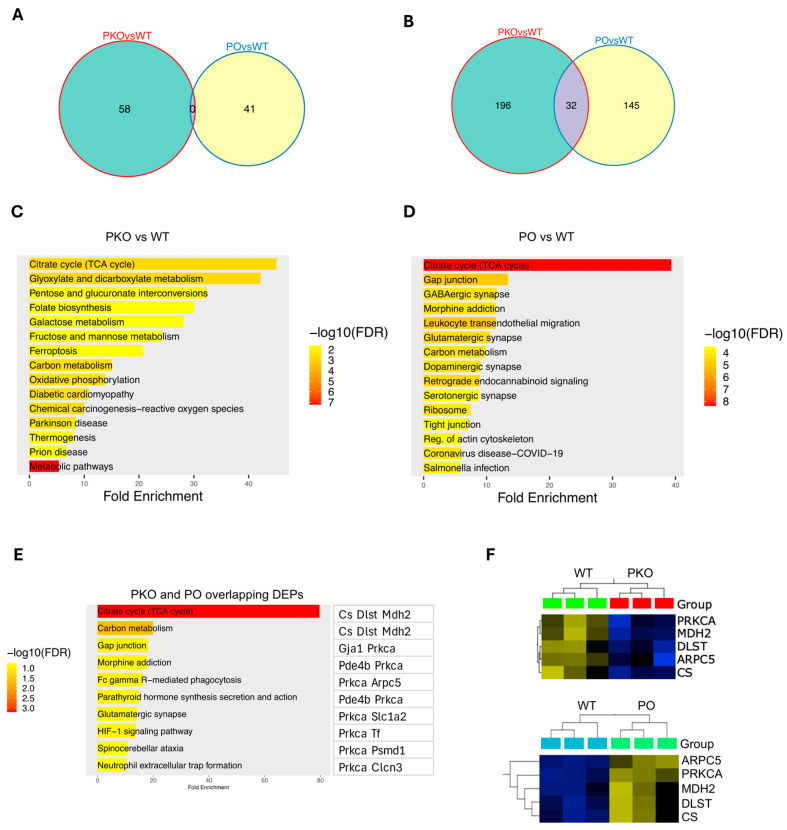
Identification of Differentially Expressed Proteins in Parkin-Deficient and Parkin-Overexpressing Rat Nucleus Accumbens. (**A**,**B**) Venn diagrams showing numbers of overlapping DEPs between PKO and PO rats detected by Qlucore Explorer at *p* < 0.01 (**A**) or *p* < 0.05 (**B**). (**A**) At *p* < 0.01, there were 58 DEPs unique to the PKOs, 41 DEPs unique to the POs, and no shared DEPs. (**B**) At *p* < 0.05, there were 196 DEPs unique to the PKOs, 145 DEPs unique to the POs, and 32 shared DEPs. (**C**) Top 20 pathways altered by parkin loss in the nucleus accumbens (*q* < 0.05, ShinyGO). (**D**) Top 20 pathways altered by parkin overexpression in the nucleus accumbens (*q* < 0.05, ShinyGO). (**E**) Pathways to which DEPs shared by PKOs and POs belong (*q* < 0.05, ShinyGO). (**F**) GSEA analysis of the shared DEPs (*p* < 0.05).

We next examined which of the other DEPs from the PKO and PO rats were changed in opposite directions. There was no overlap between 58 and 41 DEP sets at *p* < 0.01 (Figure 4A). At the *p* threshold of 0.05, there were 196 DEPs unique to the PKOs, 145 DEPs unique to the POs, and 32 shared DEPs (see Supplementary Tables S1 and S2 for the complete lists of the DEPs). (Figure 4B), with 11 DEPs changed in opposite directions (Table S7). Subsequently, we examined the pathways to which the DEPs from both groups (POs: 228 DEPs and PKO: 177 DEPS, including shared DEPs) belong, using the ShinyGo software and the KEGG knowledge base (*q* < 0.05 and a minimum of two genes/proteins per pathway). In PKO rats, the top 20 enriched pathways included multiple metabolic pathways and neurodegeneration pathways, with the most enriched pathway being the Krebs cycle (citric cycle, TCA cycle) (Figure 4C). The top 20 pathways altered by parkin overexpression in the NAc also included the Krebs cycle, carbon metabolism pathways, and pathways known to be involved in addiction, such as GABA, glutamate, and dopamine neurotransmission and regulation of actin cytoskeleton (Figure 4D). At the settings of *q* < 0.05 and a minimum of two genes per pathway, the shared DEPs belonged to 10 pathways (Krebs cycle, carbon metabolism, gap junction, morphine addiction, glutamatergic synapse, HIF-1 signaling, Fc γ R mediated phagocytosis (this process involves actin cytoskeleton), neutrophil extracellular trap formation, spinocerebellar ataxia, and parathyroid hormone synthesis secretion and action), with PRKCA and three enzymes belonging the Krebs cycle, CS, MDH2 and DLST, being the most common DEPs in these pathways (Figure 4E). To confirm the data, we ran a comparison of PKO DEPs with PO DEPs on GSEA and found CS, MDH2, DLST, PRKCA, and ARPC5 significantly changed in opposite directions in the PKO and PO NAc (*p* < 0.05, *q* < 0.05) (Figure 4F). Thus, both types of analyses, GSEA and ShinyGO, provided the same results regarding pathways and proteins significantly altered by lack or excess of parkin.

**Figure 5:**
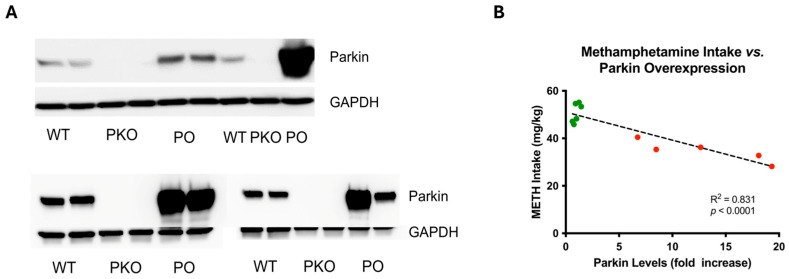
Adeno-Associated, Virus-Mediated Overexpression of Parkin in Rat Nucleus Accumbens Is Variable and Negatively Correlates with Methamphetamine Self-Administration. (**A**) Assessment of parkin levels in wild-type, parkin knockout (PKO), and parkin-overexpressing nucleus accumbens (*n* = 7) by SDS-PAGE and western blotting, with GAPDH as a loading control. The top panel shows the results from samples analyzed by proteomics, whereas the bottom panel shows the results from additional samples. Variability in parkin overexpression should be noted. (**B**) In our previous study, parkin overexpression negatively correlated with METH intake in the drug self-administration paradigm [4]. Green dots represent parkin levels in wild-type nucleus accumbens, whereas the red dots represent parkin levels in parkin-overexpressing nucleus accumbens.

In summary, both types of analyses that compared DEPs in a slightly different manner (GSEA and ShinyGO) indicated that Krebs cycle enzymes are affected by manipulation of parkin levels. To validate these findings, we assessed the levels of Krebs cycle proteins by SDS-PAGE and western blotting. The NAc samples included those assessed by label-free proteomics as well as additional PKO and PO samples (total *n* = 7/group). The rationale for increasing the sample sizes was a high variability of parkin overexpression in the NAc (Figure 5A). We observed similar variability in our previous study and found that the levels of parkin overexpression in the NAc correlated with METH intake (Figure 5B).

**Figure 6:**
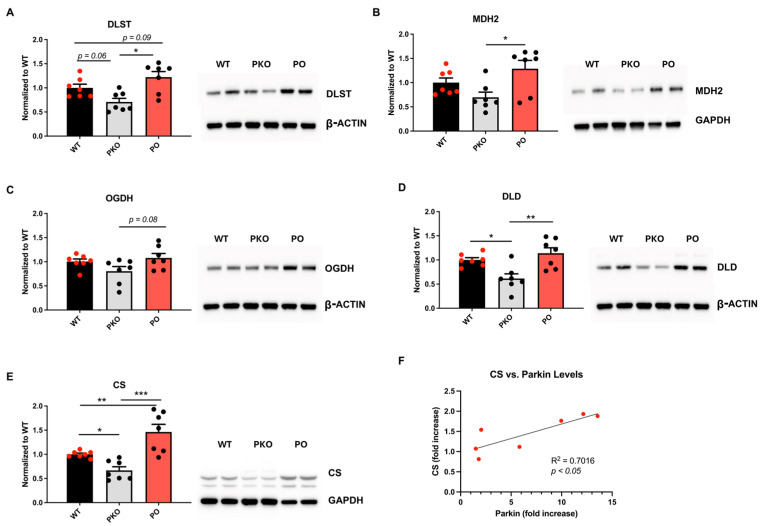
Parkin Deficit or Overexpression in Rat Nucleus Accumbens Changes Krebs Cycle Enzyme Levels in Opposite Directions. SDS-PAGE and western blotting showed statistically significant differences between parkin knockout (PKO) and parkin-overexpressing (PO) rats, as well as between these groups and wild-type (WT) controls. (**A**) dihydrolipoamide dehydrogenase (DLST), (**B**) mitochondrial malate dehydrogenase (MDH2), (**C**) 2-oxoglutarate dehydrogenase (OGDH), (**D**) dihydrolipoyl dehydrogenase (DLD), and (**E**) citrate synthase (CS). One-way ANOVA with Holm-Sidak’s post hoc test). * *p* < 0.05, ** *p* < 0.01, *** *p* < 0.001, *n* = 7/group. The data is expressed as mean ± SEM. (**F**) CS levels show a positive correlation with parkin levels (*p* < 0.05, Pearson correlation test). Abbreviations: GAPDH, glyceraldehyde 3-phosphate dehydrogenase.

The SDS-PAGE/western blotting analysis confirmed that DLST, MDH2, and CS levels decreased in the PKO NAc and increased in PO NAc (Figure 6A–C). The same trend was observed for the other two enzymes that, together with DLST, belong to the oxoglutarate dehydrogenase complex: DLD and OGDH (Figure 6D,E). The levels of CS significantly correlated with parkin levels (Figure 6F). Assessment of the levels of electron transport chain enzymes in PKO and PO rats versus their WT counterparts did not detect significant changes, except for an increase in complex IV in PO vs. PKO NAc (Supplementary Figure S2).

### 3.5. Mitochondrial Function in Parkin-Deficient and Parkin-Overexpressing Nucleus Accumbens

**Figure 7:**
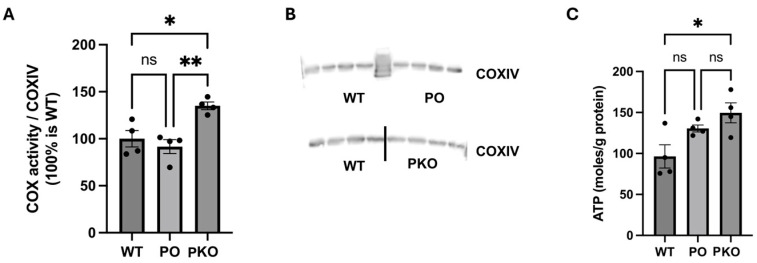
Parkin Deficit and Overexpression in Rat Nucleus Accumbens Change Mitochondrial Function. (**A**) Oxygen consumption by complex IV (COX), normalized to (**B**) the levels of subunit IV (COXIV) of complex IV measured by SDS-PAGE and western blotting. (**C**) ATP levels generated in the nucleus accumbens of wild-type (WT), parkin knockout (PKO), and parkin-overexpressing (PO) rats. * *p* < 0.05, ** *p* < 0.01, ns, not significant (one-way ANOVA with Holm–Sidak post hoc test), *n* = 4/group. The data is expressed as mean ± SEM.

To determine whether changes in the levels of mitochondrial enzymes and energy metabolism pathways lead to changes in mitochondrial function, we measured basal oxygen consumption and ATP generation. Complex IV activity and ATP production were significantly increased in the PKO NAc relative to WT NAc. Neither complex IV activity nor ATP production differed significantly in PO NAc compared to WT NAc; however, both indices showed a significant increase in PKO NAc relative to WT NAc (Figure 7).

### 3.6. Genes Coding for Differentially Expressed Proteins in Parkin-Deficient and Parkin-Overexpressing Nucleus Accumbens Overlap with Genes Associated with MUD

In our previous study [4], parkin knockout and parkin overexpression in the NAc occurred before the rats were allowed to self-administer METH. Consequently, parkin-deficit-induced or parkin-overexpression-induced changes in protein abundance in the NAc can be viewed as pre-existing changes that will counteract the upcoming effects of METH on these proteins or as changes similar to those caused by genetic factors. As the NAc is the brain area mediating the rewarding and reinforcing properties of addictive drugs [30], the changes in protein abundance induced by parkin deficit or excess in the NAc are likely contributors to the observed changes in addictive behaviors. Thus far, our data suggest that the biological processes underlying the increase and decrease in addictive behavior in PKO and PO rats, respectively, may include the Krebs cycle, stress responses, neurodegenerative pathways, signaling by glutamate and GABA neurotransmitters, actin cytoskeleton dynamics, and potentially also RNA processing. To further investigate which of the pathways found enriched in PKO and PO NAc may underlie the anti-addictive properties of parkin, we compared the list of genes coding for DEPs identified in this study (*p* < 0.05) to the list of genes that two other studies found associated with predisposition to develop MUD. One was a meta-analysis of gene association studies in humans, and the other was a transcriptome study in mice bred for high and low METH consumption [3,42]. In PKO NAc, the overlapping genes encoded antioxidant proteins, angiotensin, and phosphatidylinositol 4-kinase α. In PO NAc, only the γ-aminobutyric acid type A receptor subunit beta 2 was identified (Table 4).

### 3.7. Top Upstream Transcriptional Regulators Prediction by IPA

**Figure 8:**
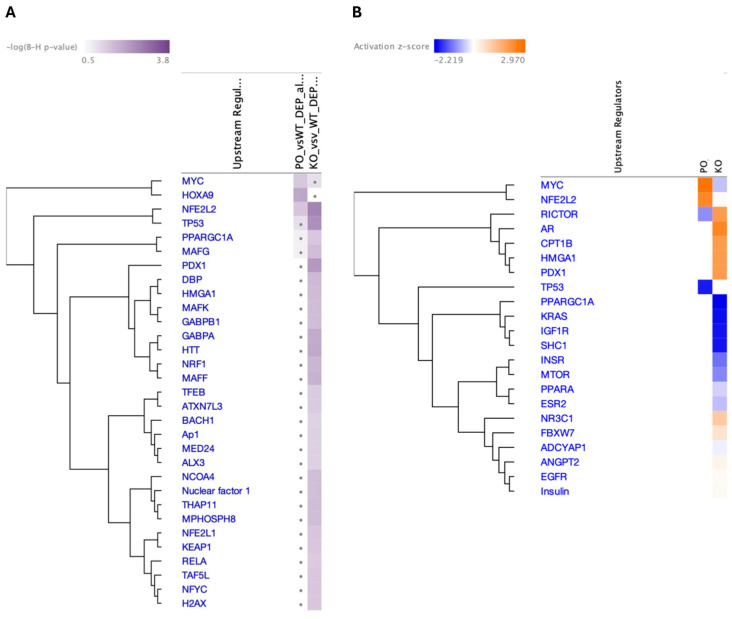
Top Upstream Transcriptional Regulators in Parkin-Deficient and Parkin-Overexpressing Rat Nucleus Accumbens. IPA-predicted upstream transcriptional regulators in the nucleus accumbens of parkin knockout (PKO) vs. wild-type (WT) and parkin-overexpressing (PO) vs. WT rats presented according to (**A**) Benjamini–Hochberg (B-H) *p*-value or (**B**) z-score, which predicts activation or inhibition state of a gene/pathway.

The predicted top three upstream transcriptional regulators in PKO NAc identified by the IPA as significantly changed (*p* < 0.05) were nuclear factor-2 (NRF2 or NFE2L2), TP53, and PDX1 (Figure 8A). Other significant transcription factors included those regulating energy metabolism, differentiation, and gene expression (Benjamini–Hochberg [B-H] *p* <0.05), including THAP11 (Ronin), a transcription factor regulating the expression of genes required for mitochondrial function, including *parkin* and genes coding for the components of the ETC. Only three transcriptional regulators were identified as significant (B-H *p* <0.05) in PO NAc: HOXA9, MYC, and NRF2. MYC was upregulated in PO NAc and downregulated in PKO NAc (Figure 8B). TP53 and PDX1 were identified as highly significant in PKO rats, while MYC and HOXA9 were identified as highly significant in PO rats.

## 4. Discussion

Our previous study in a rat model of MUD demonstrated that parkin knockout was associated with increased self-administration of METH and conditioned place preference. In contrast, overexpression of parkin in the NAc before METH exposure had the opposite effect on these METH addictive behaviors [4]. The present study investigated how parkin knockout and overexpression alter the NAc proteome relative to that of WT rats, as some of these changes may underlie the observed anti-addictive properties of parkin. This is the first study to present NAc proteomes altered by parkin loss or overexpression. In PKO rats, the top 20 enriched pathways included multiple metabolic pathways and neurodegeneration/stress response pathways, with the most enriched metabolic pathway being the Krebs cycle. The top 20 pathways altered by PO in the NAc also included the Krebs cycle and carbon metabolism pathways, as well as pathways known to be involved in addiction, such as GABA and glutamate signaling and regulation of the actin cytoskeleton. The mitochondrial Krebs cycle was one of the pathways altered in the opposite direction in PKO NAc and PO NAc. Parkin-related changes in Krebs cycle protein levels did not translate to parallel changes in basal oxygen consumption and ATP production. To narrow down the list of candidate pathways to those that could influence METH addictive behaviors in our rat model, we compared the genes coding for the DEPs identified in this study to a list of genes known to underlie predisposition to develop MUD [3,36]. Stress response genes were identified in PKO NAc, whereas a GABA receptor gene was identified in PO NAc.

Parkin has a well-established role in maintaining mitochondria in both health and disease [43]. Besides mitochondrial homeostasis, parkin regulates stress responses, actin cytoskeleton dynamics, exocytosis/endocytosis, cell survival, and the cell cycle [44,45]. There is also evidence that parkin regulates glutamatergic and dopaminergic neurotransmission [12,46]. The pathways identified as enriched by GSEA and ShinyGO analysis include these biological processes, thus aligning with the existing literature data. Many pathways found significantly enriched in PKO and PO NAc were not shared or changed in opposite directions. This finding is not surprising, considering that (i) compensatory changes likely occur in the brain during the development of PKOs and (ii) parkin was overexpressed only in NAc neurons and not throughout the whole brain. The PKO-induced compensatory changes could also explain why different sets of DEPs are the most dramatically changed in PKO and PO rats compared to their WT counterparts. We focused on pathways that changed in opposite directions by parkin knockout or overexpression because METH addictive behaviors were opposite in PKO versus PO rats in our previous study, in which manipulation of parkin levels occurred before METH self-administration [4]. Therefore, changes in protein abundance in the NAc induced by parkin deficit or overexpression can be viewed as pre-existing changes that counteract the effects of METH on these proteins or as changes like those caused by genetic predisposition to develop MUD.

IPA-identified upstream regulators confirm the involvement of parkin as a modulator of most of the identified pathways, particularly stress responses. For example, we identified NRF2 as an upstream transcriptional regulator in both parkin-deficient and parkin-overexpressing NAc. In PO NAc, NRF2 was markedly and significantly increased, whereas in PKO NAc, we found a small but significant decrease in NRF2. A marked increase in NRF2 in PO NAc supports parkin’s role as a redox molecule by providing antioxidant capacity for NAc neurons. NRF2 target genes comprise an extensive network of antioxidant enzymes (including antioxidant glutathione (GSH) system enzymes), proteins involved in xenobiotic detoxification, repair, and removal of damaged proteins, as well as inhibition of inflammation [47,48]. NRF2 also regulates mitochondrial biosynthesis [49], as does the MYC transcription factor [50], which was increased in PO NAc and decreased in PKO NAc. MYC transcription factor regulates a diverse array of cellular processes in the brain, including cell cycle, proliferation, metabolism, differentiation, and apoptosis [51]. The TP53 transcription factor responds to diverse cellular stresses to regulate the expression of target genes, thereby inducing cell cycle arrest, apoptosis, senescence, DNA repair, or changes in cellular metabolism [52]. These findings support the role of parkin in stress responses, mitochondrial homeostasis, and neurotransmission in rat NAc.

### 4.1. Parkin and Energy Metabolism in Rat Nucleus Accumbens

Parkin regulates mitochondrial biogenesis via the degradation of PARIS [53], a major transcriptional repressor of PGC-1α (peroxisome proliferator-activated receptor γ (PPARγ) coactivator-1α), a master regulator of mitochondrial biogenesis [47,53,54,55,56]. Parkin can also alter the levels of selected mitochondrial proteins by regulating local mRNAs or facilitating protein transport from mitochondria to the cytoplasm for degradation [57,58]. For example, Zheng et al. demonstrated that parkin overexpression upregulated PGC-1α and increased the transcription of multiple mitochondrial genes in mouse cortical neurons [55]. In contrast, Stevens et al. demonstrated that parkin knockout in the mouse ventral midbrain resulted in defects in mitochondrial biogenesis [59]. Our study showed that parkin knockout decreased the levels of ETC complex I and complex II subunits in the rat striatum [60,61]. Regarding the Krebs cycle, a study reported that parkin overexpression restored Krebs cycle metabolism in neurons under oxidative stress through mitochondrial biogenesis [62], whereas parkin knockout in neurons from isogenic human induced pluripotent stem cells increased Krebs cycle activity [63]. Overall, our proteomic data of energy metabolism pathways being downregulated by parkin knockout and upregulated by parkin overexpression in the NAc agrees with the previous findings. The SDS-PAGE/western blotting analysis detected deficits in Krebs cycle enzymes in PKO NAc and increases in their levels in NAc overexpressing parkin. However, the analysis did not detect significant changes in the levels of ETC enzymes in PKO or PO NAc, except for an increase in complex IV in PO NAc relative to PKO NAc. The reason for this occurring could be the ability of GSEA to detect more subtle changes in protein levels than western blotting. High variability of parkin overexpression in the NAc likely contributed to the lack of more significant changes in PO rats compared to other groups. Varying levels of parkin overexpression using the AAV2/6 vector can stem from several biological and technical factors, such as the use of a CMV promoter or WPRE in AAV vector design or the area of parkin overexpression not aligning precisely with the dissected NAc. Overall, our data suggest that mitochondrial energy metabolism proteins were only subtly decreased in PKO NAc and subtly increased in PO NAc in METH-naïve rats.

Complex IV, the terminal complex of the ETC, plays a vital role in cellular respiration and is tightly regulated [64,65,66]. It catalyzes the reduction of molecular oxygen to water, driving the proton gradient required for ATP synthesis. In contrast to our expectations, parkin knockout significantly increased complex IV activity in the NAc, whereas parkin overexpression had no effect. As a critical control point in mitochondrial respiration, complex IV activity is influenced by factors such as tissue-specific isoforms, the ATP/ADP ratio, and post-translational modifications [67]. It is possible that the overall reduction in mitochondrial content in PKO NAc led to enzyme dephosphorylation and enhanced complex IV-specific activity as a compensatory response to the reduced mitochondrial mass. A few studies reported data on basal oxygen consumption in mitochondria (i.e., the one we assessed in the present study) [68,69,70]; however, the results were inconsistent (a decrease, no change, and increase), which likely depended on the brain region assessed, the age of experimental animals, and the parkin exon manipulated to generate PKOs. For example, Giguere et al. [70] reported that primary SNc and VTA cultures from PKO mice displayed an increased oxygen consumption rate (by 50%) but reduced ATP content relative to WT controls, suggesting uncoupled mitochondria. Zanon et al. [71] found a decreased ATP production rate in DAergic SH-SY5Y cells with the knockdown of parkin. The observed increase in ATP levels in PKO NAc may stem from enhanced complex IV activity and/or the upregulation of energy pathways, such as glycolysis. Another explanation for the discrepancy in our results in PKO rat brains and those from previous studies is that different cell types were analyzed. Our study assessed a mixture of neurons and glia, and the neurons were GABAergic, not dopaminergic.

Parkin overexpression in mouse cortical neurons increased the mRNA levels of several mitochondrial proteins, including NDUFS1 and COX4. Still, it did not significantly affect the oxygen consumption rate [55]. These findings agree with our PO NAc data and suggest that parkin overexpression did not change the coupling of mitochondrial respiration to the ETC in rat NAc.

### 4.2. Parkin and Neurotransmission in Rat Nucleus Accumbens

In addition to supporting ETC function, Krebs cycle metabolites regulate chromatin modifications, DNA methylation, the hypoxic response, immunity, and glutamate metabolism [72,73]. For example, citrate, a product of CS enzyme action, promotes the synthesis of nucleotides and lipids. At the same time, α-ketoglutarate, a substrate for the oxoglutarate dehydrogenase complex—which consists of OGDH, DLST, and DLD—is a key modulator of the hypoxic response. α-Ketoglutarate can also be converted to glutamate, which, in turn, is linked to the synthesis and metabolism of GABA [73]. Succinyl coenzyme A, a product of the OGDG complex action, plays a crucial role in energy production and metabolism not only by supporting ATP production but also by participating in ketone body biosynthesis [72,74]. Consequently, the altered levels of Krebs cycle enzymes in PKO and PO NAc could lead to changes in the activities of these enzymes, followed by shifts in neuronal processes beyond energy metabolism. Only CS levels significantly correlated with parkin levels in PO NAc, suggesting a potential increase in α-ketoglutarate and altered glutamatergic and/or GABAergic neurotransmission. This notion agrees with data from biological pathways and DEP analyses. Because both neurotransmitters have been implicated in MUD [3,75] and because parkin overexpression occurred before METH self-administration in our behavioral study [4], parkin overexpression might have countered METH addiction-promoting changes in these neurotransmitter systems in our previous study.

### 4.3. Parkin and Actin Cytoskeleton Dynamics in Rat Nucleus Accumbens

Among the significantly enriched pathways, not only energy metabolism but also WASPS and WAVES, as well as the mitotic cell cycle, were significantly altered in opposite directions in PKO and PO NAc. This finding aligns with evidence that parkin regulates the levels of mitotic proteins and actin through ubiquitination [16,76]. WASPs and WAVEs promote actin polymerization through binding G-actin and the ARP2/3 complex [77]. The Arp2/3 complex, coordinated by the WASP and WAVE families, drives actin assembly during several intracellular stress response pathways [78]. Actin cytoskeleton dynamics have been linked to substance use disorders, including MUD [79] because actin mediates the trafficking of transporters and receptors such as glutamatergic AMPA receptors [26,80,81]. In post-mitotic neurons, mitotic cycle proteins play critical roles in physiological processes such as DNA repair, apoptosis, and regulation of cytoskeletal organization [40,82]. Thus, our data indicate that parkin is involved in actin cytoskeleton dynamics in rats NAc and might regulate METH-induced addictive behaviors by regulating actin cytoskeleton-mediated receptor trafficking and stress responses in the NAc.

### 4.4. Parkin and Differentially Expressed Proteins

There is no effective parkin inhibitor or activator. Consequently, identifying proteins downstream of parkin that could substitute for parkin in the biological pathways it mediates is warranted. The proteomic data indicate that parkin regulates stress responses in rat NAc. The DEPs that showed the most significant changes in abundance upon parkin knockout were ALD1, ITPA, SDC4, and BCR. ALD1 has multiple functions, including detoxifying lipid peroxidation products such as 4-hydroxy-2-nonenal (4-HNE) [83]. It also plays a role in glucose homeostasis. The primary function of ITPA is to prevent the incorporation of noncanonical purine nucleotides into RNA and DNA, including mitochondrial DNA (reviewed in [84]). Its deficiency leads to the accumulation of ITP, which may cause structural alterations in the ribosome and mistranslation. Additionally, rat brain L-glutamic acid decarboxylase, which is involved in the synthesis of GABA, is inhibited by ITP. Lastly, ITP can activate G-proteins involved in signal transduction. Thus, ITPA deficiency in PKO NAc could lead to multiple abnormalities, including stress and a deficit in GABA. BCR is a protein with a unique structure, possessing two opposing regulatory activities toward small GTP-binding proteins. It is active in glutamatergic synapses and postsynaptic densities and has been proposed to play a critical role in regulating neuronal vulnerability in response to oxygen and glucose deprivation [85]. SDC4′s (syndecan-4′s) functions at the cell membrane include stabilizing growth-factor–receptor interactions and creating a physical interface between actin and the extracellular matrix (ECM). It transmits mechanotransduction signals through the activation of PKCα and plays a role in the assembly of the actin cytoskeleton to mediate endocytosis, adhesion, and migration [86]. Its deficiency could affect all these processes in PKOs.

DEPs that most dramatically changed their abundance upon parkin overexpression were B3A3 and U520. B3A3 is a sodium-independent anion exchanger that mediates the electroneutral exchange of chloride for bicarbonate ions across the cell membranes. An increase in postsynaptic extracellular pH facilitates the activation of NMDA receptors, thereby augmenting excitatory glutamatergic transmission and inhibiting GABAergic transmission [87,88]. Therefore, it is possible that the downregulation of B3A3 in the PO NAc leads to alkaline pH in NAc terminals projecting to the ventral tegmental area (VTA) and modulates GABA transmission in the VTA in PO rats. U520 belongs to the DEXH-box family of putative RNA helicases and plays a role in pre-mRNA splicing as a core component of precatalytic, catalytic, and post-catalytic spliceosomal complexes (UniProt, the Universal Protein resource, a central repository of protein data).

DEPs found to be shared and changing in opposite directions in PKO and PO NAc included Krebs cycle enzymes (CS, MDH2, and DLST), ARPC5, UGT2B1, and PRKCA. UGT2B1 encodes UDP-glucuronosyltransferase, which is essential for the elimination and detoxification of drugs, xenobiotics, and endogenous compounds [89]. PRKCA codes for PKCα, a calcium-activated, phospholipid- and diacylglycerol (DAG)-dependent serine/threonine-protein kinase that is involved in multiple biological processes, including neuronal plasticity via regulation of glutamatergic receptor trafficking [90,91]. The protein exhibits anti-apoptotic and anti-inflammatory functions. Parkin might have altered the abundance of PKCα via regulation of the levels of its substrate PICK1, a PKCα-binding protein [92,93]. PKCα-PICK1 interaction mediates AMPA receptor trafficking [92] and, consequently, AMPA receptor-dependent neuronal plasticity, which, in turn, is involved in addiction [94]. Furthermore, the PRKCA gene was identified as altered in METH [95] and morphine addiction [96]. Thus, parkin may modulate METH addictive behaviors via modulation of neuronal plasticity.

### 4.5. Parkin and Genetics of Methamphetamine Use Disorder

Our finding of parkin deficit and excess changing specific pathways as well as METH addictive behaviors in the opposite directions does not provide evidence that these pathways underlie the addictive behaviors. Nevertheless, there is compelling indirect evidence to support this possibility. Regarding energy metabolism, METH use causes dysregulation of glucose metabolism in brain areas belonging to addiction circuitry in abstinent experimental animals and humans, decreasing glucose metabolism in most of these areas [97,98,99], including the NAc [23]. There is also evidence for reduced glucose utilization in the NAc influencing METH addictive behaviors [23,100,101,102]. Furthermore, self-administered METH dysregulates Krebs cycle function [103,104] and actin-mediated glutamatergic AMPA receptor trafficking [79]. GABAergic neurotransmission also has a role in MUD [3,42,105]. Finally, METH has been proven to cause oxidative stress and inflammation [21], while antioxidative stress and anti-inflammatory agents have been shown to attenuate the self-administration of the drug [19,106].

When we investigated which of the genes encoding the PKO DEPs overlap with genes known to underlie MUD [3,42], we found decreases in several proteins involved in stress responses: glutathione S-transferases (GSTs), cytoplasmic NAD(P)H quinone dehydrogenase 1 (NQO1), and hyaluronan and proteoglycan link protein 1 (HAPLN1). GSTs conjugate ROS, while NQO1 prevents the one-electron reduction of quinones, which results in the production of radical species. HAPLN1 is an extracellular matrix protein with antioxidant and anti-inflammatory properties [107] that has been implicated in METH addiction. For example, a reduced abundance of HAPLN1 in synapses of the prefrontal cortex and NAc was associated with long-term forced abstinence from heroin self-administration [108].

Another protein underlying MUD and downregulated in PKO NAc was angiotensinogen (AGT). AGT is a precursor of angiotensin II, which regulates blood pressure, modulates extracellular matrix components [109], and is one of the dopaminergic neurotransmission modulators [110]. Via the latter function, it can regulate METH addictive behaviors. An increase in angiotensin II via its receptor A1 (ATR1) triggers oxidative stress and inflammation in amphetamine-exposed rats [111,112]. Inhibition of ATR1 decreases METH self-administration via AT1R-PLCβ-CREB signaling pathway [113]. Angiotensin IV blocks the acquisition of METH-conditioned place preference in rats [114]. Moreover, high angiotensin II levels may even exacerbate neurodegeneration, activating the nicotinamide adenine dinucleotide phosphate (NADPH) oxidase complex and increasing ROS production [110]. As oxidative stress has a role in MUD [19], these data support the notion that reduced stress responses in PKO NAc may predispose to MUD. Furthermore, the parkin deficit slightly decreased phosphatidylinositol 4-kinase α (PI4KA). This protein acts on phosphatidylinositol (PtdIns) in the first committed step in the production of the second messenger inositol-1,4,5,-trisphosphate (IP3). Loss of parkin leads to dysregulation of IP3 receptor activity, robustly increasing ER calcium release [115]. High levels of calcium stimulate respiratory chain activity, resulting in the generation of reactive oxygen species (ROS). There is also evidence that IP3 receptor (regulated by IP3 and calcium) could mediate METH reward [116]. This data suggests dysregulation of the inositol phosphate system in PKO NAc.

In PO NAc, we found downregulation of γ-aminobutyric acid type A receptor subunit beta 2 (GABRB2). This agrees with the finding that parkin deficit increases GABAB receptors in multiple brain areas [46]. GABRB2 receptor genes were implicated in the predisposition to MUD in humans [3]. Moreover, Gabrb2 KO mice displayed less depression-like behavior than WT mice [117]. Depression can be a consequence and a predisposing factor in substance use disorders. METH impairs the signaling of GABAB receptors in the NAc which, in turn, leads to increases in dopamine release in the mesocorticolimbic pathway, while activation of these receptors decreases METH self-administration (in [118]). Consequently, decreased levels of GABRB2 protein in the NAc of PO rats could mediate, in part, attenuated METH self-administration in these rats.

## 5. Conclusions

In rat NAc, parkin modulates stress responses, actin cytoskeleton dynamics, the GABA system, and the Krebs cycle. Some of these pathways may be associated with susceptibility to developing MUD.

## 6. Limitations and Future Directions

The study has some limitations. Total body knockout of parkin is compared to parkin overexpression in one brain area. Utilizing parkin knockdown in the NAc would be a model to delineate parkin functions in the NAc without confounding compensatory changes occurring during the development. We did not assess the proteome from NAc microinjected with non-coding AAV. Therefore, although the most enriched pathways in the PO NAc were related to RNA processing, we could not determine whether parkin or AAV was involved in mRNA processing. Parkin’s overexpression was very variable, thus complicating data interpretation further. Finally, we did not assess NAc proteomes in PKO and PO rats after METH self-administration. Such a study is warranted and currently in progress.

## Figures and Tables

**Table 1:** Top enriched pathways and biological processes identified by GSEA (*p* < 0.01, *q* < 0.05) in the nucleus accumbens of parkin knockout (PKO) rats, as compared to wild-type (WT) controls.

Name	Size	Matches	ES	abs(ES)	NES	*p*	*q*
REACTOME_THE_CITRIC_ACID_TCA_CYCLE_ AND_RESPIRATORY_ELECTRON_TRANSPORT	178	74	−0.63798	0.637986	−2.3925	0	0
REACTOME_RESPIRATORY_ELECTRON_ TRANSPORT_ATP_SYNTHESIS_BY_ CHEMIOSMOTIC_COUPLING_AND_HEAT_ PRODUCTION_BY_UNCOUPLING_PROTEINS	127	44	−0.67383	0.673836	−2.2669	0	0.004754
KEGG_PARKINSONS_DISEASE	130	47	−0.63380	0.633808	−2.1624	0	0.020338
KEGG_OXIDATIVE_PHOSPHORYLATION	132	42	−0.65043	0.65043	−2.1719	0	0.022539
KEGG_HUNTINGTONS_DISEASE	182	64	−0.59313	0.59313	−2.1477	0	0.024089
GOBP_SECONDARY_METABOLIC_PROCESS	56	11	−0.85764	0.85764	−2.0945	0	0.027277
WP_ELECTRON_TRANSPORT_CHAIN_ OXPHOS_SYSTEM_IN_MITOCHONDRIA	106	37	−0.65395	0.65395	−2.1107	0	0.028262
REACTOME_RESPIRATORY_ ELECTRON_TRANSPORT	103	31	−0.67907	0.67907	−2.095	0	0.029688
WP_METAPATHWAY_ BIOTRANSFORMATION_PHASE_I_AND_II	185	24	−0.70232	0.70232	−2.1020	0	0.029817
GOBP_TRICARBOXYLIC_ACID_CYCLE	34	24	−0.70233	0.70233	−2.1139	0	0.031092
GOBP_CELLULAR_RESPIRATION	187	62	−0.58517	0.58517	−2.1213	0.001832	0.03328
GOBP_OXIDATIVE_PHOSPHORYLATION	148	46	−0.59655	0.59655	−2.0551	0	0.046927
REACTOME_PYRUVATE_METABOLISM_ AND_CITRIC_ACID_TCA_CYCLE	55	32	−0.63985	0.63985	−2.0437	0	0.047845
GOBP_ELECTRON_TRANSPORT_CHAIN	178	50	−0.59981	0.59981	−2.0451	0	0.049982

Abbreviations: GSEA, gene set enrichment analysis; ER, enrichment score; NES, normalized enrichment score.

**Table 2:** Top enriched pathways and biological processes identified by GSEA (*p* < 0.01, *q* < 0.05) in the NAc of PO rats, as compared to WT controls.

Name	Size	Matches	ES	abs(ES)	NES	*p*	*q*
GO_RNA_SPLICING_VIA_ TRANSESTERIFICATION_REACTIONS	267	45	0.71708	0.71708	2.34074	0	0
GO_RNA_SPLICING	367	52	0.695816	0.695816	2.33113	0	0
GO_RNA_PROCESSING	835	138	0.595748	0.595748	2.29978	0	0
GO_MRNA_PROCESSING	432	58	0.668237	0.668237	2.27658	0	0
GO_MRNA_METABOLIC_PROCESS	611	136	0.553283	0.553283	2.1398	0	0.000381
mRNA Splicing-Major Pathway	106	22	0.759172	0.759172	2.13459	0	0.000272
mRNA Splicing	106	22	0.759172	0.759172	2.13459	0	0.000272
mRNA Processing	156	26	0.730662	0.730662	2.13292	0	0.000211
Processing of Capped Intron-Containing Pre-mRNA	137	26	0.730662	0.730662	2.13292	0	0.000211
Gene Expression	1082	190	0.514697	0.514697	2.06178	0	0.002379
KEGG_SPLICEOSOME	128	23	0.722244	0.722244	2.05753	0	0.002162
GO_OSSIFICATION	251	33	0.642403	0.642403	1.99171	0	0.005629
GO_RIBONUCLEOPROTEIN_ COMPLEX_BIOGENESIS	440	104	0.529386	0.529386	1.97458	0	0.007684
Metabolism of mRNA	220	118	0.514172	0.514172	1.94801	0	0.010534
GO_NCRNA_PROCESSING	386	81	0.534853	0.534853	1.93834	0	0.012812
Thromboxane signaling through TP receptor	23	9	0.86424	0.86424	1.92381	0	0.015104
GO_RIBOSOMAL_SMALL_ SUBUNIT_BIOGENESIS	59	13	0.785658	0.785658	1.91994	0	0.015167
GO_SPLICEOSOMAL_COMPLEX_ASSEMBLY	53	9	0.831624	0.831624	1.91816	0.003135	0.014694
Metabolism of RNA	266	124	0.503464	0.503464	1.91488	0	0.014772
GO_OXIDOREDUCTION_COENZYME_ METABOLIC_PROCESS	107	45	0.58676	0.58676	1.90271	0	0.018743
GO_NUCLEAR_TRANSCRIBED_MRNA_ CATABOLIC_PROCESS_NONSENSE_ MEDIATED_DECAY	118	75	0.522073	0.522073	1.86156	0	0.03783
GO_OSTEOBLAST_DIFFERENTIATION	126	25	0.635636	0.635636	1.85942	0	0.037321
L13a-mediated translational silencing of Ceruloplasmin expression	104	82	0.512746	0.512746	1.85683	0	0.03548
3′-UTR-mediated translational regulation	104	82	0.512746	0.512746	1.85683	0	0.03548
Formation of a pool of free 40S subunits	94	76	0.517639	0.517639	1.85581	0	0.034441
GO_RRNA_METABOLIC_PROCESS	255	72	0.517731	0.517731	1.8484	0	0.037178
GO_RIBOSOME_BIOGENESIS	308	77	0.513105	0.513105	1.84816	0	0.035872
Nonsense-Mediated Decay	106	74	0.518657	0.518657	1.84526	0	0.035071
Nonsense Mediated Decay Enhanced by the Exon Junction Complex	106	74	0.518657	0.518657	1.84526	0	0.035071
GO_REGULATION_OF_RNA_SPLICING	97	24	0.641889	0.641889	1.83829	0.001427	0.037359
GO_TRANSLATIONAL_INITIATION	146	87	0.502547	0.502547	1.83806	0	0.03643
Signal amplification	32	13	0.746826	0.746826	1.83442	0	0.03761
GO_NCRNA_METABOLIC_PROCESS	533	88	0.501376	0.501376	1.83227	0	0.03872
Presynaptic function of Kainate receptors	21	7	0.870796	0.870796	1.83088	0	0.037241
G beta:gamma signalling through PLC beta	20	7	0.870796	0.870796	1.83088	0	0.037241
Eukaryotic Translation Initiation	112	85	0.503589	0.503589	1.82892	0	0.036488
Cap-dependent Translation Initiation	112	85	0.503589	0.503589	1.82892	0	0.036488
GTP hydrolysis and joining of the 60S ribosomal subunit	105	82	0.504021	0.504021	1.82533	0	0.038107
KEGG_CITRATE_CYCLE_TCA_CYCLE	32	23	0.65316	0.65316	1.82266	0.001364	0.039618
Eukaryotic Translation Elongation	89	72	0.509709	0.509709	1.81343	0	0.044788
Nonsense Mediated Decay Independent of the Exon Junction Complex	89	69	0.51292	0.51292	1.81174	0	0.044832
GO_RNA_CATABOLIC_PROCESS	227	84	0.500861	0.500861	1.81094	0	0.044241
Formation of the ternary complex, and subsequently, the 43S complex	48	41	0.564116	0.564116	1.80663	0	0.045756
GO_REGULATION_OF_CIRCADIAN_RHYTHM	103	19	0.670194	0.670194	1.80448	0	0.04623
Translation	148	99	0.48732	0.48732	1.80395	0	0.045435
GO_AEROBIC_RESPIRATION	53	29	0.604936	0.604936	1.8022	0	0.045854
Peptide chain elongation	84	69	0.506827	0.506827	1.80094	0	0.045465
Translation initiation complex formation	55	45	0.550771	0.550771	1.79833	0	0.045279
Activation of the mRNA upon binding of the cap-binding complex and eIFs, and subsequent binding to 43S	56	45	0.550771	0.550771	1.79833	0	0.045279
GO_TRICARBOXYLIC_ACID_METABOLIC_ PROCESS	37	26	0.630346	0.630346	1.79827	0	0.044412
ADP signalling through P2Y purinoceptor 1	25	9	0.801737	0.801737	1.79777	0	0.043989
GO_CELLULAR_RESPIRATION	143	58	0.535198	0.535198	1.79474	0	0.045668
Eukaryotic Translation Termination	84	68	0.509707	0.509707	1.79437	0	0.045004
GO_RIBONUCLEOPROTEIN_COMPLEX_ LOCALIZATION	118	18	0.661641	0.661641	1.79191	0.001458	0.045738
KEGG_RIBOSOME	88	66	0.50964	0.50964	1.78599	0	0.048781

Abbreviations: GSEA, gene set enrichment analysis; ER, enrichment score; NES, normalized enrichment score.

**Table 3:** Enriched pathways and biological processes that have been downregulated by parkin knockout and upregulated by parkin overexpression in rat nucleus accumbens.

Name	Group	Size	Matches	ES	abs(ES)	NES	*p*	*q*
GOBP_AEROBIC_RESPIRATION	PKO	86	39	−0.571	0.571	−1.899	0.00385	0.1238
GOBP_AEROBIC_RESPIRATION	PO	86	39	0.564	0.564	1.781	0	0.0516
GOBP_CELLULAR_RESPIRATION	PKO	187	62	−0.585	0.585	−2.121	0.00183	0.0333
GOBP_CELLULAR_RESPIRATION	PO	187	62	0.519	0.519	1.802	0	0.0467
GOBP_ENERGY_DERIVATION_BY_ OXIDATION_OF_ORGANIC_ COMPOUNDS	PKO	278	79	−0.516	0.516	−1.953	0	0.0835
GOBP_ENERGY_DERIVATION_BY_ OXIDATION_OF_ORGANIC_ COMPOUNDS	PO	278	79	0.454	0.454	1.628	0.00524	0.2103
GOBP_POSITIVE_REGULATION_OF_ MITOTIC_CELL_CYCLE	PKO	118	15	−0.657	0.657	−1.786	0.00986	0.2058
GOBP_POSITIVE_REGULATION_OF_ MITOTIC_CELL_CYCLE	PO	118	15	0.652	0.652	1.672	0.00884	0.1433
GOBP_TRICARBOXYLIC_ACID_CYCLE	PKO	34	24	−0.702	0.702	−2.114	0	0.0311
GOBP_TRICARBOXYLIC_ACID_CYCLE	PO	34	24	0.618	0.618	1.806	0.00145	0.0456
KEGG_CITRATE_CYCLE_TCA_CYCLE	PKO	31	23	−0.689	0.689	−2.022	0.00197	0.0528
KEGG_CITRATE_CYCLE_TCA_CYCLE	PO	31	23	0.653	0.653	1.877	0	0.0263
REACTOME_RHO_GTPASES_ACTIVATE_ WASPS_AND_WAVES	PKO	36	20	−0.638	0.638	−1.813	0.01158	0.1819
REACTOME_RHO_GTPASES_ACTIVATE_ WASPS_AND_WAVES	PO	36	20	0.652	0.652	1.807	0.00287	0.0453
WP_TCA_CYCLE_AKA_KREBS_OR_ CITRIC_ACID_CYCLE	PKO	18	14	−0.773	0.773	−2.028	0.00190	0.0573
WP_TCA_CYCLE_AKA_KREBS_OR_ CITRIC_ACID_CYCLE	PO	18	14	0.632	0.632	1.621	0.02025	0.2179

Abbreviations: GSEA, gene set enrichment analysis; ER, enrichment score; NES, normalized enrichment score. KEGG, GOPB, and WP are pathway databases. Red is upregulation while green is downregulation.

**Table 4:** List of genes coding for identified DEPs overlapping with known genes underlying MUD.

Symbol *	Entrez Gene Name	Gene Symbol—Rat	FC_PKO	*p*-Value PKO
AGT	angiotensinogen	Agt	1.699	0.00153
GSTO1	glutathione S-transferase omega 1	Gsto1	−1.139	0.0226
GSTP1	glutathione S-transferase pi 1	Gstp1	−1.235	0.00321
HAPLN1	hyaluronan and proteoglycan link protein 1	Hapln1	−1.943	0.0428
NQO1	NAD(P)H quinone dehydrogenase 1	Nqo1	−1.593	0.00605
PI4KA	phosphatidylinositol 4-kinase α	Pi4ka	−1.044	0.00694
Symbol *	Entrez Gene Name	Gene Symbol—Rat	FC_PO	*p*-value PO
GABRB2	γ-aminobutyric acid type A receptor subunit beta 2	Gabrb2	−1.813	0.0191

* Human Gene symbols. Abbreviations: FC, fold change; PKO, parkin knockout; PO, parkin overexpression. Red color denotes upregulation whereas blue color denotes downregulation.

## Data Availability

The authors confirm that the data supporting the findings of this study are available within the article and its Supplementary Materials. The mass spectrometry proteomics data have been deposited to the ProteomeXchange Consortium via the PRIDE [1] partner repository with the dataset identifier PXD065059.

## Supplementary Materials

The following supporting information can be downloaded at https://www.mdpi.com/article/10.3390/biom15070958/s1. Figure S1. Assessment of the purity of the mitochondrial fraction from the rat nucleus accumbens. Figure S2. Assessment of protein levels of four electron transport chain enzymes in the rat nucleus accumbens. Table S1. List of proteins detected in parkin-deficient (PKO) and wild-type (WT) nucleus accumbens by label-free proteomics. Table S2. Differentially Expressed Proteins (DEPs) in parkin-deficient nucleus accumbens as compared to wild-type control. Table S3. List of proteins detected in parkin-overexpressing (PO) and wild-type (WT) nucleus accumbens by label-free proteomics. Table S4. Differentially Expressed Proteins (DEPs) in parkin-overexpressing nucleus accumbens as compared to wild-type control. Table S5. PKO vs WT. Table S6. PO vs WT. Table S7. Genes encoding the 32 DEPs shared by parkin-deficient (PKO) and parkin-overexpressing (PO) nucleus accumbens (*p* < 0.05, *q* < 0.05).
