# Supplementary material for: The Proteomic Landscape of Parkin-Deficient and Parkin-Overexpressing Rat Nucleus Accumbens: An Insight into the Role of Parkin in Methamphetamine Use Disorder
Source: Biomolecules. 2025 Jul 3;15(7):958. doi: 10.3390/biom15070958 (PMC12292523; doi:10.3390/biom15070958)
Supplement: Supplementary file 1 [file biomolecules-15-00958-s001.zip › Original blots_Moszczynska.pdf]

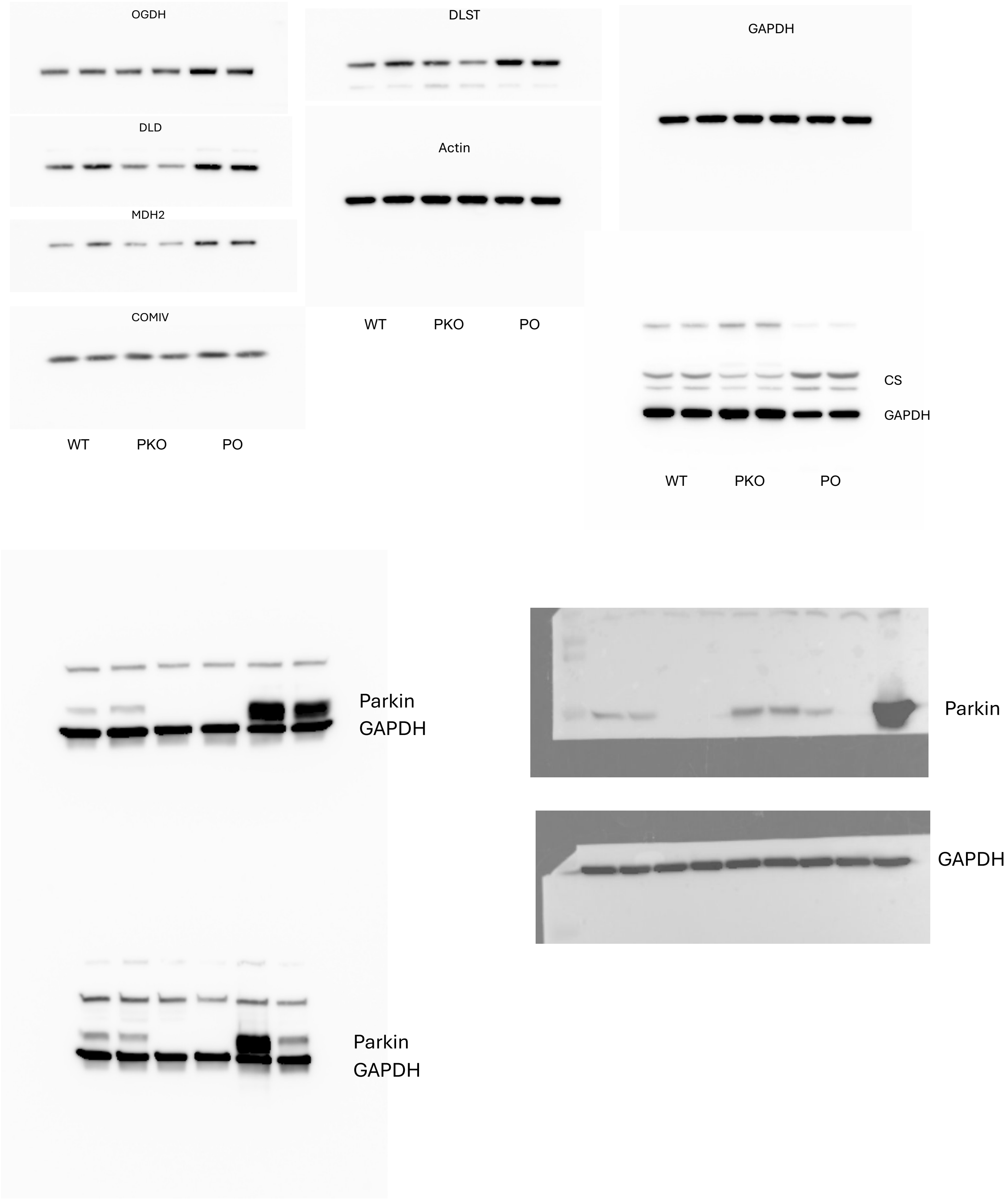

Original blots for Figures 5 and 6. After the SDS-PAGE, the membranes were cut horizontally into several pieces and incubated with different primary antibodies against Krebs cycle enzymes, parkin, and the loading controls. This approach saved nucleus accumbens samples. Some proteins with similar molecular weights were incubated with their respective primary antibodies sequentially, using stripping and reblotting techniques. Actin and GAPD were detected last.

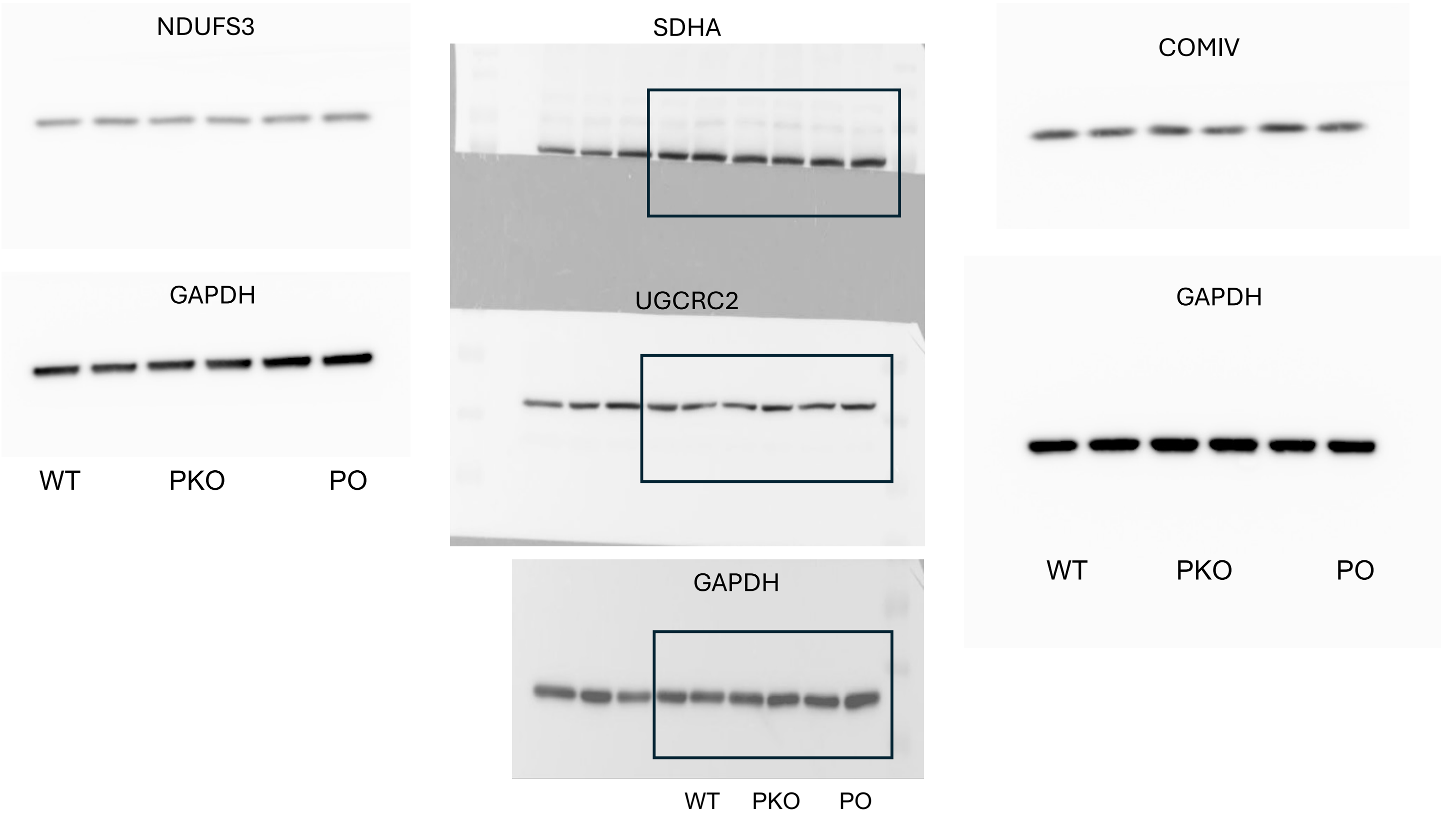

Original blots for Supplementary Figure 2. After the SDS-PAGE, the membranes were cut horizontally into pieces and incubated with different primary antibodies against ETC cycle enzymes and the loading control GAPDH.
