# Supplementary material for: The Proteomic Landscape of Parkin-Deficient and Parkin-Overexpressing Rat Nucleus Accumbens: An Insight into the Role of Parkin in Methamphetamine Use Disorder
Source: Biomolecules. 2025 Jul 3;15(7):958. doi: 10.3390/biom15070958 (PMC12292523; doi:10.3390/biom15070958)
Supplement: Supplementary file 1 [file biomolecules-15-00958-s001.zip › Revised Suppl. Data2_Final.pdf]

Supplementary Data

The proteomic landscape of parkin-deficient and parkin-overexpressing rat nucleus accumbens: an insight into the role of parkin in methamphetamine use disorder

Akhil Sharma [1], Tarek Atasi [1], Florine Collin [2], Weiwei Wang [2], TuKiet T. Lam [2,3,4], Rolando Garcia Milian [5], Tasnim Arroum [6], Lucynda Pham [6], Maik Hüttemann [6] , and Anna Moszczynska [1,*]

[1] Department of Pharmaceutical Sciences, Wayne State University, Detroit, MI 48202, USA
[2] Department of Molecular Biophysics and Biochemistry, Yale University, New Haven, CT 06520, USA
[3] Keck Mass Spectrometry & Proteomics Resource, Yale School of Medicine, New Haven, CT 06520, USA
[4] Yale/NIDA Neuroproteomics Center, Yale School of Medicine, New Haven, CT 06520, USA
[5] Harvey Cushing/John Hay Whitney Medical Library, Yale University, New Haven, CT 06520, USA
[6] Center for Molecular Medicine and Genetics, Wayne State University, Detroit, MI 48201, USA
* Correspondence: amosz@wayne.edu

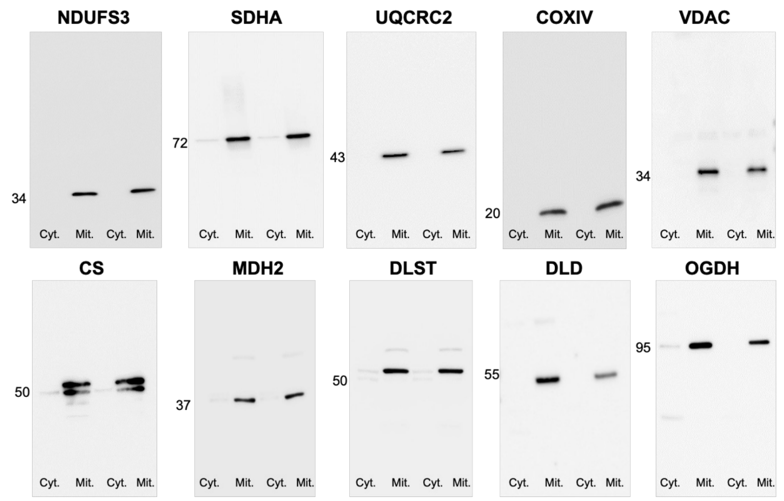

Figure S1. Assessment of the purity of the mitochondrial fraction from the rat nucleus accumbens. Mitochondria were isolated from the rat nucleus accumbens. The mitochondrial and cytosolic fractions were analyzed using SDS-PAGE under reducing conditions. Membranes were probed with antibodies specific to electron transport chain enzyme subunits (NDUFS3, complex I subunit; SDHA, complex II subunit; UQCRC2, complex III subunits; COXIV, complex IV subunit), five Krebs cycle enzymes (CS, MDH2, DLST, DLD, and OGDH), and Voltage-Dependent Anion Channels (VDAC). The mitochondrial enzyme bands were detected at the correct molecular weights (numbers on the left side of the blots represent molecular weights in kilodaltons [kDa]). No bands were detected in the cytosolic fraction. Abbreviations: CS, citrate synthase; MDH2, mitochondrial malate dehydrogenase; DLST, dihydrolipoamide S-succinyltransferase; DLD, dihydrolipoamide dehydrogenase; OGDH, 2-oxoglutarate dehydrogenase; Cyt, cytosol; Mit., mitochondria.

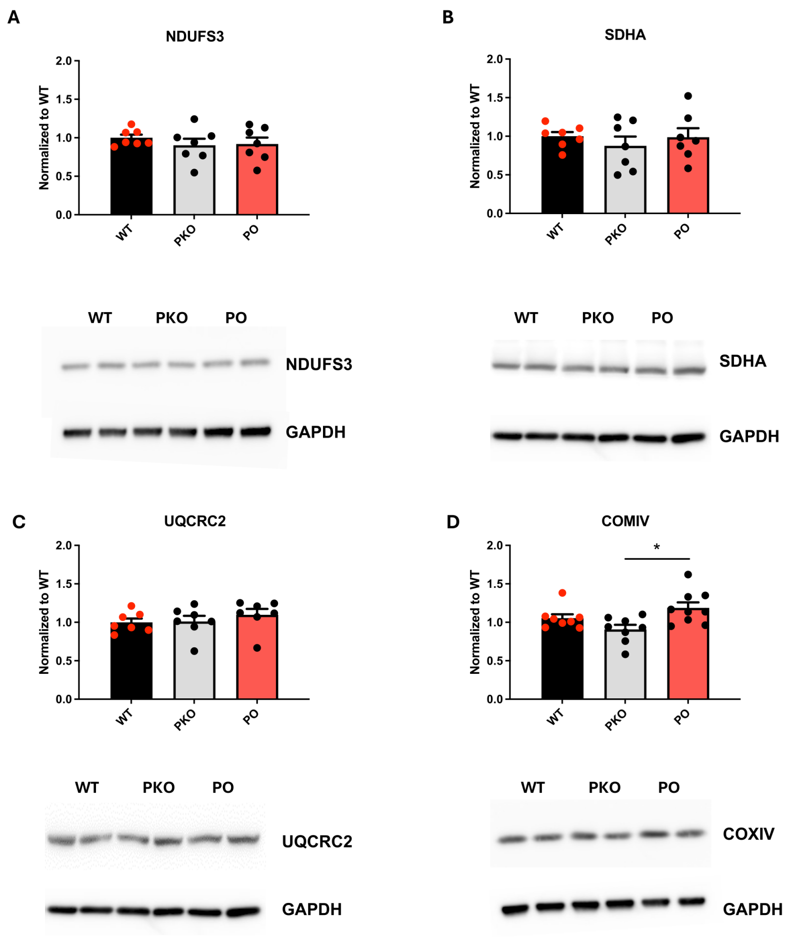

Figure S2. Assessment of protein levels of four electron transport chain enzymes in the rat nucleus accumbens. The SDS-PAGE and western blot analysis revealed no statistically significant differences between parkin knockout (PKO) and parkin overexpressing (PO) rats, nor between these groups and wild-type (WT) controls, except for the difference in complex IV levels between the PKO and PO groups (*$p < 0.05$, one-way ANOVA with Holm-Sidak's post hoc test). **(A)** NDUFS3, complex I subunit; **(B)** SDHA, complex II subunit; **(C)** UQCRC2, complex III subunits; **(D)** COXIV, complex IV subunit); GAPDH was utilized as the loading control.
